# Outcome of Sentinel Hospital-based and CDC-based ART Service Delivery: A Prospective Open Cohort of People Living with HIV in China

**DOI:** 10.1038/srep42637

**Published:** 2017-02-14

**Authors:** Chuanyi Ning, Kumi M. Smith, Chase D. McCann, Fengyu Hu, Yun Lan, Fuchun Zhang, Hao Liang, Jinmin Zhao, Joseph D. Tucker, Weiping Cai

**Affiliations:** 1 Guangzhou Eighth People’s Hospital, Guangzhou Medical University, Guangzhou, Guangdong, China; 2 The University of North Carolina Project-China, Guangzhou, Guangdong, China; 3 Department of Infection Disease, University of North Carolina at Chapel Hill, Chapel Hill, North Carolina, USA; 4 Medical Scientific Research Center & Guangxi Key Laboratory of AIDS Prevention and Treatment, Guangxi Medical University, Guangxi, China; 5 Johns Hopkins Bloomberg School of Public Health, Baltimore, Maryland, USA; 6 Department of Microbiology & Immunology, University of North Carolina at Chapel Hill, Chapel Hill, North Carolina, USA

## Abstract

The primary objective of this study was to obtain insights into the outcomes of people living with HIV who accessed services through HIV/AIDS sentinel hospital-based and ART service delivery in China. Post-hoc analyses of an open cohort from an observational database of 22 qualified HIV/AIDS sentinel hospital-based and two CDC-based drug delivery facilities (DDFs) in Guangdong Province was completed. Linkage to care, mortality and survival rates were calculated according to WHO criteria. 12,966 individuals received ART from HIV/AIDS sentinel hospitals and 1,919 from DDFs, with linkage to care rates of 80.7% and 79.9%, respectively (P > 0.05). Retention rates were 94.1% and 84.0% in sentinel hospitals and DDFs, respectively (P < 0.01). Excess mortality was 1.4 deaths/100 person-years (95% CI: 1.1, 1.8) in DDFs compared to 0.4 deaths/100 person-years (95% CI: 0.3, 0.5) in hospitals (P < 0.01). A Cox-regression analysis revealed that mortality was much higher in patients receiving ART from the DDFs than sentinel hospitals, with an adjusted HR of 3.3 (95% CI: 2.3, 4.6). A crude HR of treatment termination in DDFs was 7.5 fold higher (95% CI: 6.3, 9.0) compared to sentinel hospitals. HIV/AIDS sentinel hospital had better retention, and substantially lower mortality compared to DDFs.

Over the past two decades the number of people receiving antiretroviral therapy (ART) on the global scale has been steadily increasing by a large margin each year^1^,^2^. With the continuous growth in the number of people living with HIV/AIDS, the tasks of treating and managing follow-up visits becomes increasingly difficult. This is especially true for low and middle-income countries (LMIC) where HIV prevalence is often higher and the number of adequately trained medical personnel is limited. Additionally, people living in LMIC face multiple structural challenges to medication adherence including lack of transportation, as well as the social organizations involved in response. The promise of HIV treatment as prevention has focused attention on how to best implement treatment practices with many cluster-randomized trials investigating competing strategies (HPTN 078, 071, 065)^3^,^4^,^5^. Despite these initial suggestions of success, the barriers to implementation of test-and-treat strategies need to be more fully evaluated.

Numerous countries around the world have formulated and improved relevant policies concerning publicity, education, comprehensive intervention, and follow-up visit management for HIV treatment^6^,^7^,^8^. In sub-Saharan Africa, increased funding made available through the Global Fund to Fight AIDS, Tuberculosis and Malaria and the President’s Emergency Plan for AIDS Relief (PEPFAR) has helped focus intervention strategies on patients’ day-to-day adherence to ART^9^,^10^. In the United States, a recent study revealed that 72.8% of HIV-infected patients received care at facilities supported by the Ryan White HIV/AIDS Program^11^. Although many countries have significantly improved access to ART programs, achieving and maintaining a satisfactory long-term retention rate remains probelmatic^12^. It has been suggested that programs such as these, combined with adequate health facility infrastructure, can help serve patients with multiple social determinants of poor health and have been associated with improved outcomes^13^.

The process of ART delivery in China, however, is very region specific, owing to its size and epidemiological heterogeneity as well as the imbalance of economic autonomy^14^,^15^. Since beginning a pilot combination antiretroviral therapy (cART) program among former plasma donors in 2002, China has rapidly scaled up the National Free Antiretroviral Treatment Program (NFATP) in Center for Disease Control (CDC)-based ART service delivery facilities^16^. Chinese health authorities are expanding treatment-based prevention strategies, partially based on evidence that ART is already slowing the incidence of HIV infections in China^17^,^18^,^19^. In China, 90% of HIV-infected patients receiving ART treatment were linked to at least one free testing of viral load, and more than 50% of patients had already accessed free medicine through ART service delivery facilities^20^. By the end of 2014, more than 295,358 patients across the country had received free antiretroviral treatment^20^. However, implementation of this treatment model was driven by thousands of CDC-based ART service delivery facilities focused solely on drug delivery, where delegation of physician tasks to less well-trained staff has hampered the effectiveness of antiretroviral therapy. In contrast, Guangdong Province has established a system of Sentinel hospitals in which patients wishing to receive HIV treatment through the NFATP are cared for by highly trained Infectious Disease specialists.

As the Chinese government has emphasized the importance of comprehensive HIV treatment and treatment-as-prevention strategies, health systems research on decentralizing HIV services is essential to achieving this goal^21^. Therefore, it is important to assess if variations in drug delivery models impact relative effectiveness of treatment-as-prevention based strategies. Here we examine two ART delivery models currently used within the Guangdong Province of China to determine the extent to which such differences should be factored into policy. To evaluate patients’ outcome and retention rate of HIV/AIDS Highly Active ART (HAART) program on HIV-infected patients in the sentinel hospital and drug delivery facilities (DDFs), we analyzed data on mortality, immunologic response status, and risk factors of all previously treatment naive adult patients enrolled in the China NFATP of Guangdong province between 2005–2014.

## Methods

### Study Design and Setting

Data for this study were obtained through the NFATP observational database, described previously^2^. All patients with at least one visit to 24 health care facilities who initiated antiretroviral therapy from January 2005 through 30 June 2014 were included in this analysis. Patients were eligible for inclusion in this study if they had ever received ART through the NFATP and were 18 years of age or older at treatment initiation. At all sites, patients testing positive for HIV were provided with antiretroviral treatment counseling in the clinic. Patients were excluded if they did not start triple drug therapy, had missing treatment regimen information, or used ART outside of national guidelines. Patients who initiated treatment but failed to return for scheduled appointments were contacted and reason for termination was recorded. If, after multiple attempts, patients could not be contacted they were recorded as loss of follow-up. Treatment facility transfers and death were determined based upon linking patient unique Identity Number. The main outcomes evaluated were mortality and retention status, while risk factors associated with death were also evaluated in this study and were calculated according to World Health Organization (WHO) criteria.

### Ethics Statement

The institutional review board of the Guangzhou Eighth People’s Hospital approved this analysis, according to the international and Chinese ethical guidelines. All the methods and the data analysis protocols were carried out in accordance with the WHO approved guidelines. Informed consent was obtained from all subjects prior to enrollment in this cohort.

### Definition of HIV/AIDS sentinel hospitals and DDFs

Based on WHO guidelines and expert consensus on HIV/AIDS care in China, we defined AIDS sentinel hospitals according to the following criteria: (1) hospitals specific for infectious disease treatment or general hospitals with a department of infectious diseases, including traditional medicine hospitals, (2) the department of infectious diseases should have at least two physicians (at least one infectious disease fellow) and 3 nurses, all of whom were qualified by the Ministry of Health or Health Office of Clinical Study for the treatment of people living with HIV and (3) hospitals should have an HIV screening laboratory, HIV screening techniques, blood testing, liver function and kidney function testing equipment and technology, x-ray inspection equipment and techniques, opportunistic infections testing equipment and technology. Since 2005, 22 of the professional or general hospitals in Guangdong province qualified as HIV/AIDS sentinel hospitals, and two chronic disease centers which lead by the Center for Disease Control of Guangdong were defined as drug delivery facilities (DDFs). The latter served multiple functions of prevention, monitoring, and control of chronic and infectious diseases led by the CDC of Guangdong. The two different treatment facility models were all based on the NFATP guidelines described previously^22^,^23^. In Guangdong province, during ART in sentinel hospitals, CD4 cell count testing was completed every 6 months, and plasma viral load was quantified at least once per year at Guangzhou Eighth People’s Hospital. In addition, hepatic function was monitored at months 0, 0.5, 1, 2, 3, 6 of cART, and every 6 months thereafter. Laboratory results were compared at baseline as well as at the last follow up visit for participants in sentinel hospitals and ART service delivery health facilities. Geographic location was taken into consideration as a possible factor of death and drug stoppage after initiation of ART.

### Statistical Analysis

Data collected included demographic characteristics, current symptoms, laboratory results, treatment regimen start and stop dates and the reason for treatment change or termination. We compared baseline characteristics between cohorts using the Mann-Whitney test for continuous variables, because none fulfilled the Kolmogorov-Smirnov test for normality. We used the Pearson chi-square statistic for dichotomous and categorical variables. We used Cox proportional hazards modeling to assess hazard ratio (HR) estimated potential risk factors. We entered covariates that we predetermined to be clinically significant into full multivariate Cox models. We calculated survival curves by using life tables (SPSS, Chicago, Illinois) and assessed statistical significance between groups using the log-rank test because the assumption of proportionality was fulfilled. We used SPSS, version 19.0, SAS, version 9.2 (SAS Institute, Cary, NC), and ArcGIS version 10.2 (Esri, Redlands, CA) for all analyses. All hypothesis testing was 2-sided, with a level of 0.05 considered significant.

## Results:

### Demographic Characteristics

**Figure 1:**
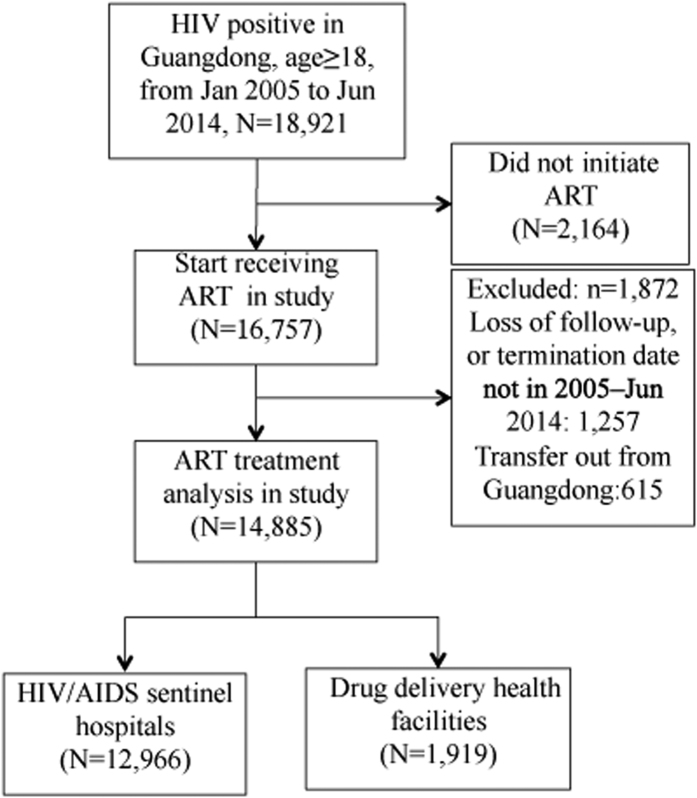
Flow chart index tests in patients included in sentinel hospital-based and CDC-based ART Service Delivery in the study.

Of the 18,921 HIV patients seen at one of the 22 HIV/AIDS sentinel hospitals or 2 ART delivery health facilities in Guangdong Province between January 2005 and June 2014, 16,757 (88.6%) initiated cART. Of those, 1,872 were excluded from the analysis because of loss to follow up; 1,257 because there was no follow up visit within the study dates, and 615 because the patient transferred to a different province to receive ART. Among the 14,885 patients included in this analysis, 12,966 (87.1%) received treatment at a sentinel hospital and 1,919 (12.9%) received treatment at a CDC DDF (Fig. 1).

Overall, the mean age of participants was 38.5 years, 73.2% were men, 55.6% were infected through heterosexual transmission, 19.1% through injection drug use (IDU), 18.4% through male-to-male homosexual transmission, and 29.2% had baseline symptoms within the last 3 months. Univariate analysis showed significance differences were observed between the two groups (P < 0.05) in WHO Clinical Stage of HIV/AIDS, CD4 cell count, prior treatment for Tuberculosis (TB) before ART, Hepatitis C Virus (HCV) co-infection or current use of Cotrimoxazole (Table 1). DDFs had a slightly higher percentage of females (28.1% vs 26.6%) and a significantly higher proportion of participants with HCV co-infection (26.8% vs 8.0%, P < 0.01) compared to sentinel hospitals. The mean age of patients seen in sentinel hospitals (38.2 ± 11.7) was lower, although not significantly, than those seen at DDFs (41.0 ± 11.6), however mortality occurred at a significantly younger age in the DDFs than the sentinel hospitals (40.9 ± 10.8 vs 42.6 ± 13.2, P < 0.01; Table 2).

During the last follow up visit, immunologic response improved greatly after initiating therapy, especially in patients receiving ART management in sentinel hospitals. For example, the interquartile range (IQR) of CD4 cell count at baseline was 172 (IQR, 57–261), and 369 (IQR, 250–497) at the last visit in sentinel hospital. This is in contrast to DDFs where baseline was 160 (IQR, 59–258), and increased to only 264 (IQR, 175–392) at the last visit (369 vs 264, P < 0.01). Although viral load was not significantly different between these two sites, management of antiretroviral adverse effects, based upon usage of hemodlastase, glutamic-oxalacetic transaminase, and glutamic-pyruvic transaminase, was better at sentinel hospitals than at DDFs (Table 3).

### Linkage to care and retention rate in sentinel hospitals and DDFs

Among the 18,921 HIV patients seen in Guangdong Province, 16,074 (85.0%) were diagnosed in sentinel hospitals, 2,403 (12.7%) were diagnosed in DDFs, and 444 (2.3%) were diagnosed outside of Guangdong. As a result, linkage to care was 80.7% (12,966/16,074) in sentinel hospitals and 79.9% (1,919/2,403) in DDFs, respectively (P > 0.05).

Of those included in this analysis, 479 terminated treatments because of adverse effects to medication (11.5%), patient request (21.7%), poor adherence (61.6%), or other (5.3%). Retention rates were therefore 84.0% and 94.1% in DDFs and sentinel hospitals, respectively (P < 0.01). Crude HR of drug stopping in CDC health facilities was 7.5 (95% CI: 6.3, 9.0) compared to sentinel hospitals (Table 4).

### Long-term outcomes of sentinel hospitals and DDFs

**Figure 2:**
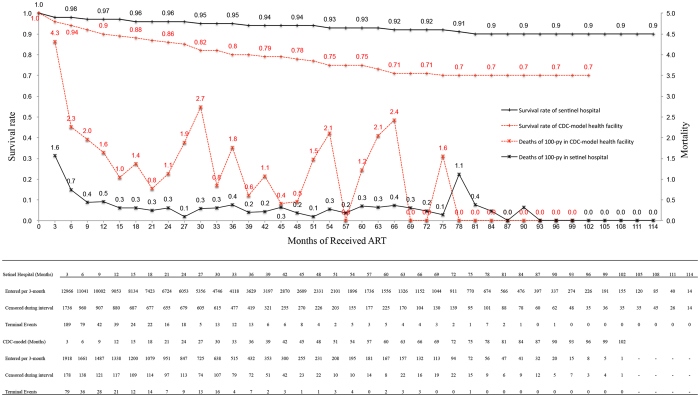
Overall excess mortality and survival rates between sentinel hospitals and CDC drug delivery health facilities. Overall change over time by mortality and life table survival rate. Mortality rate shown reflects each 3-month interval.

**Figure 3:**
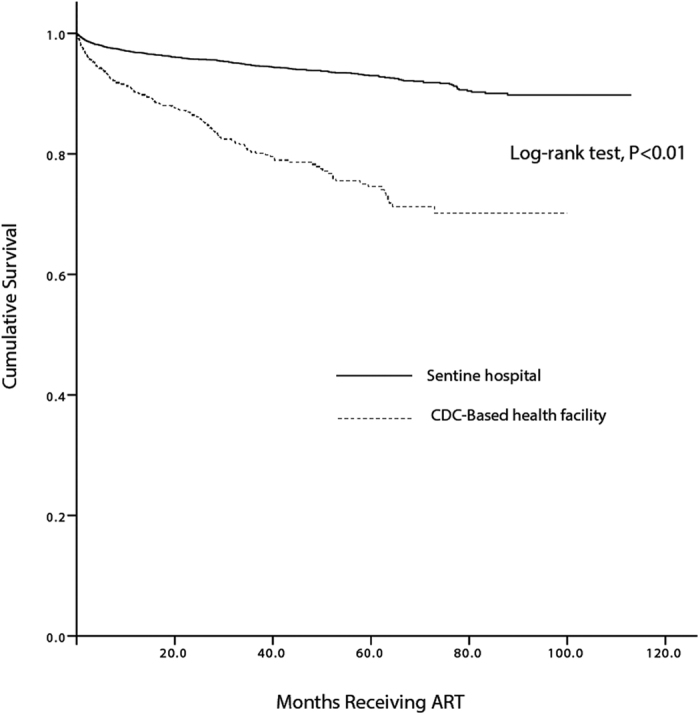
Kaplan–Meier analysis in sentinel hospital and drug deliver health facilities. Life table survival stratified by sentinel hospitals and CDC drug delivery health facilities after treatment initiation. Log-rank test, P < 0.001.

The median follow up times in DDFs and sentinel hospitals were 24.1 months (IQR, 7.1–31.2) and 28.1 months (IQR, 7.4–35.5), respectively. Of the 805 who died, 70.1% of those deaths were AIDS related, and 25.1% died from other complications. The overall excess mortality rate was 0.8 deaths/100 person-years (95% CI: 0.6, 1.0). Excess mortality was 1.4 deaths/100 person-years (95% CI: 1.1, 1.8) in DDFs compared to 0.4 deaths/100 person-years (95% CI: 0.3, 0.5) in sentinel hospitals (Fig. 2) Kaplan-Meier analysis showed that the average survival time was 78.7 (95% CI, 76.2, 81.2) months in the DDFs group compared to sentinel hospitals at 105.3 (95% CI: 104.6, 106.1) months. As seen in Figure 3, mortality was much higher in patients receiving ART in the DDFs model (Fig. 3).

### Risk factors related to death in Guangdong during long-term HAART

Gender, age at beginning HAART, baseline WHO stage and CD4 cell count, with complications of TB, HBV and Hepatitis C Virus (HCV) had unequally average unadjusted HR range from 1.3–9.6 times more risk of death. Those receiving treatment in a DDF after recruitment were 3.6 (95% CI: 3.1–4.2) times more at risk of death than those receiving treatment at a sentinel hospital (P < 0.01). Patients with a baseline CD4 cell count between 200–350 cells/ml had a higher risk of death compared to those with CD4 count >350 cells/ml, however, this was not statistically significant.

In an adjusted Cox regression analysis, the strongest risk factors for death were having a nadir CD4 cell count less than 50 cells/ml and receiving ART in DDFs. Those with CD4 cell counts less than 50 cells/ml had an adjusted HR of 6.3 (95% CI, 2.2 to 20.0) compared to those with CD4 cell counts of 350 cells/ml or greater. Adjusted HR for those treated at a DDF was 3.3 (95% CI: 2.3, 4.6) compared to those treated in a sentinel hospital. Patients with HBV, HCV and TB baseline complications had adjusted HR of 1.7 (95% CI, 1.3, 2.4), 3.0 (95% CI, 2.8, 5.2) and 1.6 (95% CI, 1.0, 2.0) respectively, compared to those without respective co-infections (Table 5).

### Geographic distributions of HIV infections and treatment sites in Guangdong province

**Figure 4:**
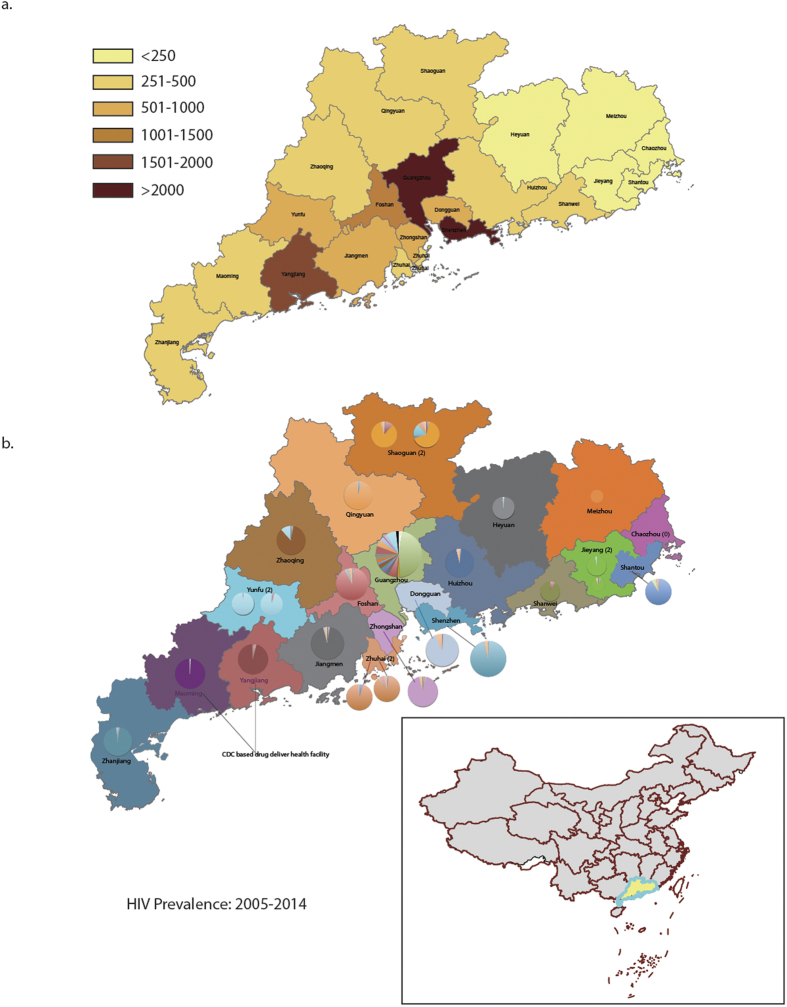
Geographic distribution of HIV infections in Guangdong Province of China from 2005–2014. (**a**) Geographic distribution of HIV prevelance in Guangdong. (**b**) Map representing patients seeking HIV treatment in other cities within Guangdong. Each city is represented by a unique color. Pie charts illustrate the percentages of patients receiving ART at each treatment facility with the color of the pie piece coorelating to the patients’ city if origin. All maps were generated using ArcGIS version 10.2 (http://www.esri.com/software/arcgis/arcgis-for-desktop/free-trial).

A general overview of HIV reported from different cities in Guangdong illustrates HIV infection has a very specific geographic pattern. The highest rates of infection were seen in the Pearl River Delta and Yuexi regions with more than half of Guangdong’s HIV infections (51.0%) coming from the Pearl River Delta (Fig. 4a). Figure 4b illustrates the imbalance between geographic distributions of reported infections and health care facilities in Guangdong province. For example, the capital city of Guangzhou and the Special Economic Zone of Shenzhen accounted for 31.2% of all infections reported from 2005–2014 and are responsible for 32.1% of patients’ care in all health care facility, yet 46.6% (2,227/4,781) of patients were receiving ART management from outside of Guangzhou. In contrast to other cities, these health care facilities are usually responsible for 65.5%-100% of local patients’ care, respectively (Fig. 4b).

## Discussion

Our findings that patients receiving treatment from DDFs had an adjusted HR of mortality of 3.3 (95% CI: 2.3, 4.6) as well as an unadjusted HR of stopping treatment of 7.5 (95% CI: 6.3, 9.0) compared to sentinel hospitals is cause for concern. The NFATP has significantly reduced the mortality rate among former blood and/or plasma donors partly because of their ease of management through the CDC-based DDFs or community hospitals in rural treatment settings despite having limited laboratory-monitoring^24^. Obviously, drugs stop is not only an indicator for ART evaluation but is also one of the barriers to achieving treatment-based prevention goals. The Guangdong province HIV/AIDS sentinel hospitals have modeled a system of integrated diagnosis, counseling and treatment instead of the national CDC leading health facilities, to help improve access and linkage to care, thereby increasing patient retention and adherence to ART regimens. However, investigations conducted in other provinces found that healthcare workers in general health facilities still face challenges other than providing professional care for patients^25^,^26^.

This study directly compared two different HIV treatment models in Guangdong and revealed that the sentinel hospital model could improve patient outcomes and reduce treatment barriers. The observed mortality rate of 1.4 deaths/100 person-years in DDFs in Guangdong is similar to results from resource wealthy areas of the general population in nine industrialized countries (0.8–1.4 deaths/100 person-years)^27^. Additionally, the overall mortality rate in Guangdong, regardless of treatment sites, was 0.8 deaths/100 person-years, which is much lower than that observed in most Chinese national survey studies (4–6 deaths/100 person-years)^28^,^29^. We believe the early success of ART in sentinel hospitals of Guangdong province calls for optimism. This experience demonstrates that it is possible, given proper resources and local government commitment, to treat many thousands of people in China’s urban settings. Studies have identified factors that reliably predict non-adherence to ART including demographic characteristics, illness experiences, social relations and so on. After adjusting for differences in patient characteristics, patients receiving care at funded facilities were more likely to achieve viral suppression (1.1 [1.0–1.2])^11^. Proponents of a test-and-treat strategy for HIV prevention are aware of the importance and formidable challenges posed by poor engagement in HIV care. Nevertheless, levels of these indicators may differ among population groups with HIV/AIDS.

Our findings support results from most previous studies, showing that people with HIV who have hepatitis co-infection have an increasing risk of death after starting antiretroviral treatment in the first month^30^. With the support of NFATP and other funding agencies, the Chinese government has demonstrated that the roll out of HIV care and treatment services in urban primary care sites is feasible on a large scale while maintaining favorable patient outcomes. Despite the burgeoning availability of ART, HIV prevention remains critical in urban China, where each year an estimated 100,000 adults and children become infected and the same amount increasing to meet criteria for ART initiation. By the end of 2014, there were 3,952 antiretroviral treatment institutions in 2,312 counties/districts within 31 provinces, autonomous regions or municipalities around the country^20^. Therefore, besides Guangdong, there are nearly 1,000 hospitals and health facilities in each province in Guangxi, Henan and Yunan respectively. Here, policymakers and funders of health care need to develop more concrete steps to strengthen the management of delegating physician-level tasks to less qualified staff when planning for the amount and cost of the hospital-based treatment regimens required in the hospital from CDC. Looking at the change in CD4 cell count at ART initiation within each treatment site may be a better way to determine treatment success in getting patients treated earlier. But due to the observational nature of the database, it is unclear if the late ART initiation in our study was the result of late HIV diagnosis, delayed enrollment into HIV care, or late ART initiation despite timely enrolment in care. In conclusion, the median CD4 cell count at ART initiation was low in our cohort, however this did increase by the last follow up visit.

Low retention in CDC-based DDFs or community hospitals was likely in part the result of less qualified healthcare workers dispensing medications. The prevalence of HIV infection and number of patients seeking care varies greatly by region as seen in a recent study reporting variation by geographic location within Guangxi China^31^. More and more patients with advanced disease may have attended higher-level facilities, such as sentinel hospitals, for treatment and ART initiation, as they are generally perceived to offer better health services than community hospitals^32^. In light of the results mentioned above, a scale up of the sentinel hospital model could enhance linkage to supportive care, reduce treatment barrier, increase CD4 follow-up management, and expand CD4 baseline coverage, thereby improving overall patient outcomes. Additional research, however, is still needed to develop interventions aimed at increasing the balance of ART management in the different sentinel hospitals.

In this study, we compared the long-term outcomes of sentinel hospital-based and CDC-based ART delivery systems in the developed province in China. As far as we know, this is the first cohort in China to compare the two different types of facilities and show that treatment in sentinel hospitals has the potential to improve patient outcomes and reduce treatment barriers. Improving ART management continues to remain a major public health challenge in China. Our findings add to the understanding of comprehensive management and control of HIV in developing countries, after the start of HAART more than 20 years ago. One limitation of this study is the inability to delineate the independent contribution of, for example, poor retention in care, delayed initiation of antiretroviral therapy, and poor adherence to antiretroviral therapy, when the same individuals are prone to deficits in all of these areas. We have made best estimates from available data, but these are further limited by our cross-sectional representation of the longitudinal process of engagement in care. Additionally, this study is focused on the spectrum of engagement in HIV care in the Guangdong province as a resource-rich setting. Obstacles to diagnosis of HIV infection, linkage to and retention in HIV care, and receipt of and adherence to antiretroviral therapy in resource-limited settings such as African countries are of critical importance to the global HIV epidemic and deserve their own evaluation. Our results may not be directly generalizable across the country. These data are from a resource-rich province where patient acuity levels may not be representative of their respective national HIV programs. We have described our data as being different from the national averages, but recognize the immigration variations across the participating treatment sites. Overall, our study has highlighted important differences in patient outcomes between the two different HIV treatment model systems in Guangdong, China. We believe further efforts should be made to adopt a sentinel hospital-based system of integrated HIV care and treatment.

## Additional Information

**How to cite this article:** Ning, C. *et al*. Outcome of Sentinel Hospital-based and CDC-based ART Service Delivery: A Prospective Open Cohort of People Living with HIV in China. *Sci. Rep.* **7**, 42637; doi: 10.1038/srep42637 (2017).

**Publisher's note:** Springer Nature remains neutral with regard to jurisdictional claims in published maps and institutional affiliations.

## Figures and Tables

**Table 1:** Baseline Patient Characteristics.

	Total N = 14885	Sentinel hospital (%) N_1_ = 12966	DDFs (%) N_2_ = 1919	χ^2^ (F)	P
Age	38.5 ± 11.7	38.2 ± 11.7	41.0 ± 11.6	6.410	0.011
18–44	11237 (75.5)	9826 (75.8)	1411 (73.6)	19.147	0.000
45–60	2591 (17.4)	2266 (17.5)	325 (16.9)		
>60	1056 (7.1)	874 (6.7)	182 (9.5)		
Sex				2.008	0.156
Man	10901 (73.2)	9522 (73.4)	1379 (71.9)		
Woman	3984 (26.8)	3445 (26.6)	539 (28.1)		
Marital status					
Unmarried	4031 (27.1)	3662 (28.2)	369 (19.2)	128.183	0.000
Divorce/widowed	1572 (10.6)	1258 (9.7)	314 (16.4)		
Married	9186 (61.7)	7972 (61.5)	1214 (63.3)		
Unknown	96 (0.6)	75 (0.6)	21 (1.1)		
Transmission Route					
Blood	203 (1.4)	200 (1.5)	3 (0.2)	1693.445	0.000
Injection drug users	2849 (19.1)	1855 (14.3)	994 (51.8)		
Men who have sex with men	2744 (18.4)	2727 (21.0)	17 (0.9)		
Heterosexual	8277 (55.6)	7468 (57.6)	809 (42.2)		
Mother to child	7 (0.0)	7 (0.1)	0 (0.0)		
Unknown	805 (5.4)	710 (5.5)	95 (5.0)		
TB, PPT (+)	1127 (7.6)	1005 (7.8)	122 (6.4)	21.851	0.000
Treat TB before ART	1048 (93.0)	947 (7.3)	101 (5.3)		
Un-treat TB before ART	79 (7.0)	58 (0.4)	21 (1.1)		
Symptoms within 3 months					
No	9203 (61.8)	8107 (62.5)	1096 (57.1)	56.750	0.000
Unknown	1330 (8.9)	1202 (9.3)	128 (6.7)		
Yes	4352 (29.2)	3658 (28.2)	694 (36.2)	141.160	0.000
Fever > 37 °C> 1mon	2072 (13.9)	1831 (14.1)	241 (12.6)		
Diarrhea > 1mon	645 (4.3)	557 (4.3)	88 (4.6)		
Oral hairy leukoplakia	525 (3.5)	383 (3.0)	142 (7.4)		
Skin lesions	682 (4.6)	505 (3.9)	177 (9.2)		
Thrush	451 (3.0)	352 (2.7)	99 (5.2)		
Herpes zoster	907 (6.1)	783 (6.0)	124 (6.5)		
Current symptoms					
Yes	5569 (37.4)	4541 (35.0)	1028 (53.6)	245.537	0.000
WHO Stage				12.167	0.000
I–III	12094 (81.2)	10597 (81.7)	1497 (78.1)		
IV (AIDS)	2691 (18.1)	2291 (17.7)	400 (20.9)		
Cotrimoxazole	3485 (23.4)	3190 (24.6)	295 (15.4)	79.423	0.000
Previously used	11255 (75.6)	9698 (74.8)	1557 (81.2)	80.906	0.000
Currently use	3249 (21.8)	2991 (23.1)	258 (13.5)		
Never used	236 (1.6)	201 (1.6)	35 (1.8)		
CD4 cell count				27.078	0.000
<50	3535 (23.7)	3064 (23.6)	471 (24.6)		
50–199	5012 (33.7)	4281 (33.0)	731 (38.1)		
200–350	4861 (32.7)	4295 (33.1)	566 (29.5)		
>350	777 (5.2)	702 (5.4)	75 (3.9)		
HBV (+)	1532 (10.3)	1319 (10.2)	213 (11.1)	1.555	0.212
HCV (+)	1548 (10.4)	1034 (8.0)	514 (26.8)	634.720	0.000

**Table 2:** Baseline Patient Characteristics Stratified by Mortality.

	Alive			P	Dead			P
	Total alive (%) N = 14080	Sentinel hospital (%) N_1_ = 12431	DDFs (%) N_2_ = 1649		Total death (%) n = 805	Sentinel hospital (%) n_1_ = 536	DDFs (%) n_2_ = 269	
Age	38.3 ± 11.7	38.0 ± 11.6	41.0 ± 11.7	0.198	42.0 ± 12.5	42.6 ± 13.2	40.9 ± 10.8	0.000
18–44	10689 (75.9)	9482 (76.3)	1207 (73.2)	0.000	548 (68.1)	344 (64.2)	204 (75.8)	0.003
45–60	2433 (17.3)	2150 (17.3)	283 (17.2)		158 (19.6)	116 (21.6)	42 (15.6)	
>60	957 (6.8)	798 (6.4)	159 (9.6)		99 (12.3)	76 (14.2)	23 (8.6)	
Sex				0.001				0.020
Man	10226 (72.6)	9084 (73.1)	1142 (69.3)		675 (83.9)	438 (81.7)	237 (88.1)	
Woman	3854 (27.4)	3347 (26.9)	507 (30.7)		130 (16.1)	98 (18.3)	32 (11.9)	
Marital status				0.000				0.179
Unmarried	3861 (27.4)	3557 (28.6)	304 (18.4)		170 (21.1)	105 (19.6)	65 (24.2)	
Divorce/widowed	1463 (10.4)	1189 (9.6)	274 (16.6)		109 (13.5)	69 (12.9)	40 (14.9)	
Married	8673 (61.6)	7619 (61.3)	1054 (63.9)		513 (63.7)	353 (65.9)	160 (59.5)	
Unknown	83 (0.6)	66 (0.5)	17 (1.0)		13 (1.6)	9 (1.7)	4 (1.5)	
Transmission Route				0.000				0.000
Blood	190 (1.3)	188 (1.5)	2 (0.1)		13 (1.6)	12 (2.2)	1 (0.4)	
Injection drug users	2461 (17.5)	1660 (13.4)	801 (48.6)		388 (48.2)	195 (36.4)	193 (71.7)	
Men have sex with men	2733 (19.4)	2716 (21.8)	17 (1.0)		11 (1.4)	11 (2.1)	0 (0)	
Heterosexual	7934 (56.3)	7195 (57.9)	739 (44.8)		343 (42.6)	273 (50.9)	70 (26.0)	
Mother to child	7 (0.0)	7 (0.0)	0 (0)		0 (0)	0 (0)	0 (0)	
Unknown	755 (5.4)	665 (5.3)	90 (5.5)		50 (6.2)	45 (8.4)	5 (1.9)	
TB, PPT (+)	1007 (7.2)	904 (7.3)	103 (6.3)	0.046	120 (14.9)	101 (19.2)	19 (7.3)	0.000
Treat TB before ART	936 (92.9)	849 (93.9)	87 (84.5)	0.000	112 (93.3)	98 (97.0)	14 (73.7)	0.000
Un-treat TB before ART	71 (7.1)	55 (6.1)	16 (15.5)		8 (6.7)	3 (3.0)	5 (26.3)	
Symptoms within 3 months								
No	8950 (63.6)	7966 (64.1)	984 (59.7)		253 (31.4)	141 (26.3)	112 (41.6)	0.000
Unknown	1204 (8.6)	1099 (8.8)	105 (6.4)		126 (15.7)	103 (19.2)	23 (8.6)	
Yes	3926 (27.9)	3366 (27.1)	560 (34.0)	0.000	426 (52.9)	292 (54.5)	134 (49.8)	
Fever > 37 °C > 1mon	1829 (13.0)	1648 (13.3)	181 (11.0)	0.000	243 (30.2)	183 (34.1)	60 (22.3)	
Diarrhea > 1mon	555 (3.9)	494 (4.0)	61 (3.7)		90 (11.2)	63 (11.8)	27 (10.0)	
Oral hairy leukoplakia	461 (3.3)	354 (2.8)	107 (6.5)		64 (8.0)	29 (5.4)	35 (13.0)	
Skin lesions	627 (4.5)	472 (3.8)	155 (9.4)		55 (6.8)	33 (6.2)	22 (8.2)	
Thrush	398 (2.8)	311 (2.5)	87 (5.3)		53 (6.6)	41 (7.6)	12 (4.5)	
Herpes zoster	808 (5.7)	723 (5.8)	85 (5.2)		99 (12.3)	60 (11.2)	39 (14.5)	0.000
Current symptoms								
Yes	5013 (35.6)	4177 (33.6)	836 (50.7)	0.000	556 (69.1)	364 (67.9)	192 (71.4)	0.000
WHO Stage								
I–III	11586 (82.3)	10251 (82.5)	1335 (81.0)	0.198	508 (63.1)	346 (64.6)	162 (60.2)	0.291
IV (AIDS)	2410 (17.1)	2110 (17.0)	300 (18.2)		281 (34.9)	181 (33.8)	100 (37.2)	
Cotrimoxazole	3273 (23.2)	3010 (24.2)	263 (15.9)	0.000	212 (26.3)	180 (33.6)	32 (11.9)	0.000
Previously used	10678 (75.8)	9351 (75.2)	1327 (80.5)	0.000	577 (71.7)	347 (64.7)	230 (85.5)	0.000
Currently use	3061 (21.7)	2832 (22.8)	229 (13.9)	0.000	188 (23.4)	159 (29.7)	29 (10.8)	0.000
Never used	212 (1.5)	180 (1.4)	32 (1.9)	0.027	24 (3.0)	21 (3.9)	3 (1.1)	0.027
CD4 cell count								
<50	3150 (22.4)	2794 (22.5)	356 (21.6)	0.006	385 (47.8)	270 (50.4)	115 (42.8)	0.000
50–199	4736 (33.6)	4122 (33.2)	614 (37.2)		276 (34.3)	159 (29.7)	117 (43.5)	
200–350	4795 (34.1)	4257 (34.2)	538 (32.6)		66 (8.2)	38 (7.1)	28 (10.4)	
>350	771 (5.5)	698 (5.6)	73 (4.4)		6 (0.7)	4 (0.7)	2 (0.7)	
HBV (+)	1463 (10.4)	1275 (10.3)	188 (11.4)	0.002	69 (8.6)	44 (8.2)	25 (9.3)	0.845
HCV (+)	1436 (10.2)	986 (7.9)	450 (27.3)	0.000	112 (13.9)	48 (9.0)	64 (23.8)	0.000

**Table 3:** Baseline Patient Characteristics Stratified by Mortality and Compared to Last Follow up Visit.

	Total Baseline^§^	Total alive	Alive (IQR)	CDC-based DDFs	Total death	Death (IQR)	CDC-based DDFs	Last follow-up visit^¶^ (IQR)		
	(IQR)		Sentinel hospital			Sentinel hospital		Total last visit	Sentinel hospital	CDC-based DDFs
CD4 (copies/μl)	164 (51, 257)	170 (57, 261)	172 (57, 261)	160 (59, 258)	45 (15, 120)*	34 (13, 103)	66 (21, 150)	360 (239, 489)**	369 (250, 497)	264 (175, 392)
CD8 (copies/μl)	746 (492, 1065)	753 (501, 1068)	745 (497, 1057)	809 (531,1173)	570 (321, 971)**	465 (269, 831)	738 (478, 1108)	828 (605,1123)*	827 (608, 1117)	845 (571, 1266)
Viral load (log)	5.1 (4.5, 5.6)	5.1 (4.5, 5.6)	5.2 (4.54, 5.65)	4.8 (4.2, 5.3)	5.2 (4.9, 5.5)	5.2 (4.6, 5.56)	5.2 (4.9, 5.2)	3442^#^ (89.3)	3191 (92.4%)	251 (62.6%)
White blood cells (10^9^/L)	4.8 (3.8, 6.1)	4.8 (3.8, 6.0)	4.8 (3.8, 6.0)	4.9 (3.9, 6.2)	4.5 (3.4, 6.5)*	4.4 (3.1, 6.3)	4.9 (3.8, 6.7)	5.7 (4.7, 6.9)*	5.6 (4.6, 6.8)	5.8 (4.7, 7.3)
Lymphocyte (10^9^/L)	1.4 (1.0, 1.9)	1.4 (1.0, 2.0)	1.4 (1.0, 1.9)	1.5 (1.1, 2.1)	1.0 (0.6, 1.5)**	0.9 (0.5, 1.4)	1.2 (0.8, 1.6)	1.9 (1.5, 2.4)*	1.9 (1.5, 2.4)	1.9 (1.5, 2.5)
Plastocyte (10^9^/L)	184 (146, 227)	184 (146, 227)	185 (147, 226)	181 (140, 233)	183 (127, 244)	182 (124, 247)	184 (134, 235)	213 (177, 253)*	214 (178, 253)	207 (162, 257)
Hemoglobin (g/L)	129 (112, 145)	130 (113, 146)*	131 (113, 146)	127 (113, 143)	109 (92, 126)*	106 (90, 125)	115 (99, 130)	140 (127, 152)*	141 (128, 152)	134 (120, 148)
Serum creatinine (μmol/L)	72.9 (61.0, 85.0)	73.0 (61.0, 84.9)	72.9 (61.0, 84.4)	73.4 (62.0, 87.0)	71.2 (57.0, 86.8)*	71.2 (56.1, 85.3)	72.0 (59.0, 89.0)	76.0 (65.0, 88.2)*	76.0 (65.2, 88.0)	77.0 (64.0, 91.0)
Blood urea nitrogen (mmol/L)	4.1 (3.2, 5.0)	4.1 (3.2, 5.0)	4.1 (3.3, 5.0)	4.0 (3.2, 4.9)	3.9 (3.0, 5.2)*	3.9 (3.0, 5.3)	4.0 (3.0, 5.2)	4.4 (3.6, 5.3)	4.4 (3.7, 5.3)	4.4 (3.6, 5.4)
Triglyceride (mmol/L)	1.3 (1.0, 1.9)	1.3 (0.95, 1.86)	1.31 (0.96, 1.89)	1.25 (0.9, 1.7)	1.3 (1.0, 1.8)	1.4 (1.0, 1.9)	1.27 (0.93, 1.70)	1.6 (1.1, 2.5)*	1.6 (1.1, 2.6)	1.4 (1.0, 2.1)
Cholesterol (mmol/L)	4.0 (3.4, 4.7)	4.0 (3.4, 4.7)	4.0 (3.4, 4.7)	4.1 (3.4, 4.8)	3.5 (2.8, 4.2)**	3.5 (2.8, 4.3)	3.5 (2.9, 4.1)	4.6 (4.0, 5.3)*	4.6 (4.0, 5.3)	4.6 (4.0, 5.3)
Blood glucose (mmol/L)	5.2 (4.7, 5.7)	5.2 (4.7, 5.7)	5.1 (4.7, 5.6)	5.3 (4.8, 5.8)	5.2 (4.7, 5.8)	5.2 (4.7, 5.8)	5.1 (4.7, 5.6)	5.4 (5.0, 5.9)*	5.4 (5.0, 5.9)	5.5 (5.1, 5.9)
Hemodlastase (U/L)	69 (55, 89)	69 (55, 88)	68 (54, 86)	78 (60, 101)	72 (53, 99)**	70 (53, 100)	76 (56, 97.5)	69 (54, 88)	68 (53, 87)	83 (65, 102)
Glutamic-oxalacetic transaminase (U/L)	27 (21, 39)	27 (21, 38)	26 (20, 37)	32 (24, 46)	36 (26, 56)**	36 (25, 53)	38 (26, 62)	25 (20, 33)*	24 (19, 32)	33 (25, 49)
Glutamic-pyruvic transaminase (U/L)	24 (16, 40)	24 (16, 39)	24 (16, 39)	28 (18, 46)	28 (18, 45)**	28 (19,45)	27 (16, 47)	25 (17, 38)	24 (17, 37)	31 (22, 51)
Total bilirubin (μmol/L)	9.7 (7.0, 13.2)	9.7 (7.0, 13.1)	9.7 (7.0, 13.1)	9.5 (6.7, 13.2)	9.3 (6.5, 13.8)*	8.8 (6.3, 13.1)	10.2 (6.9, 15.5)	8.7 (6.6, 11.4)**	8.8 (6.7, 11.5)	7.7 (5.7, 10.5)

Notice: 1. ^§^ Compare baseline between alive and death; ^¶^ compare to follow up and total baseline; 2. *P < 0.05, **P < 0.005. 3. ^#^For the viral load at time of follow up, we reported changes to the proportion of those with undetectable VL (NA, <40) to show the effectiveness of ART.

**Table 4:** Long-Term Patient Outcomes Compared between Sentinel Hospitals and DDFs.

Treatment sites$	Number of initiated ART (%)	On-therapy (%)	Death			Drug stop		
			Number of death (%)	Crude HR of Death (95% CI)	P	Number of drug stop (%)	Crude HR of drug stop (95% CI)	P
HIV/AIDS sentinel hospital	12966 (87.1)	12195 (89.7)	536 (4.1)	1.0	–	235 (1.8)	1.0	—
CDC-based DDFs	1919 (12.9)	1406 (10.3)	269 (14.0)	3.6 (3.1, 4.2)	0.000	244 (12.7)	7.5 (6.3, 9.0)	0.000

^$^The median follow up times in DDFs and sentinel hospitals were 24.1 months (IQR, 7.1–31.2) and 28.1 months (IQR, 7.4–35.5), respectively.

**Table 5:** Rates and Risk Factors for Death or Treatment Failure by Cox Proportional Hazards Regression.

	HR	95%CI	P	HRadj	95% CI	P
Age						
18–44	1.0					
45–60	1.3	1.1–1.6	0.002	2.1	1.5–3.0	
>60	2.3	1.9–2.9	0.000	2.5	1.3–4.1	0.001
Sex						
Female	1.0					
Male	2.2	1.8–2.7	0.000	1.4	1.0–2.0	0.010
HBV	1.0					
	2.0	1.5–2.7	0.000	1.7	1.3–2.4	0.001
HCV						
	3.7	2.9–4.8	0.000	3.0	2.2–4.3	0.000
TB	1.0					
	2.3	1.9–2.8	0.000	1.6	1.1–2.4	0.000
WHO						
I–III	1.0					
IV (AIDS)	2.3	2.0–2.7	0.000	1.6	1.1–2.1	0.005
CD4						
>350	1.0					
200–350	1.6	0.7–3.7	0.265	1.3	0.4–4.4	0.480
50–199	5.2	2.3–11.7	0.000	3.4	1.1–10.9	0.000
<50	9.6	4.3–21.6	0.000	6.3	2.0–20.0	0.000
Treatment sites						
Sentinel hospital	1.0					
CDC-based drug delivery health facility	3.6	3.1–4.2	0.000	3.3	2.3–4.6	0.000
